# 2-Azido-2-de­oxy-3,4-*O*-isopropyl­idene-2-*C*-methyl-d-talono-1,5-lactone

**DOI:** 10.1107/S160053681001500X

**Published:** 2010-04-30

**Authors:** Sarah F. Jenkinson, Ni Dai, George W. J. Fleet, David J. Watkin

**Affiliations:** a Department of Organic Chemistry, Chemistry Research Laboratory, University of Oxford, Mansfield Road, Oxford OX1 3TA, England; b Department of Chemical Crystallography, Chemistry Research Laboratory, University of Oxford, Mansfield Road, Oxford OX1 3TA, England

## Abstract

The relative stereochemistry of the title compound, C_10_H_15_N_3_O_5_, was confirmed by the crystal structure determin­ation. The absolute configuration was determined from the use of d-lyxonolactone as the starting material. The six-membered ring adopts a boat conformation with the larger azide group, rather than the methyl group, in the bowsprit position. In the crystal structure, a bifurcated inter­molecular O—H⋯O/O—H⋯N hydrogen bond links mol­ecules into chains running parallel to the *b* axis.

## Related literature

For carbohydrates as chirons, see: Lichtenthaler & Peters (2004 ▶); Fechter *et al.* (1999 ▶); Fleet (1989 ▶). For branched sugars and their use as chirons, see: Rao *et al.* (2008 ▶); Jones *et al.* (2008 ▶); Booth *et al.* (2008 ▶); Hotchkiss, Kato *et al.* (2007 ▶); da Cruz *et al.* (2008 ▶); Soengas *et al.* (2005 ▶). For the structures of similar sugars, see: Chesterton *et al.* (2006 ▶); Booth *et al.* (2007 ▶); Hotchkiss, Jenkinson *et al.* (2007 ▶); Baird *et al.* (1987 ▶); Bruce *et al.* (1990 ▶); Punzo *et al.* (2005 ▶). For the extinction correction, see: Larson (1970 ▶).
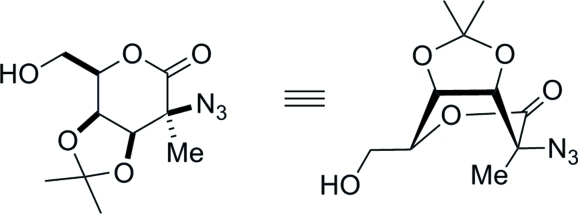


## Experimental

### 

#### Crystal data

C_10_H_15_N_3_O_5_
*M* *_r_* = 257.25
Orthorhombic, 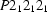

*a* = 5.9481 (3) Å
*b* = 13.3427 (7) Å
*c* = 15.6351 (9) Å
*V* = 1240.86 (12) Å^3^
*Z* = 4
Mo *K*α radiation
μ = 0.11 mm^−1^
*T* = 150 K
0.20 × 0.15 × 0.05 mm

#### Data collection

Nonius KappaCCD diffractometer
Absorption correction: multi-scan (*DENZO*/*SCALEPACK*; Otwinowski & Minor, 1997 ▶) *T*_min_ = 0.89, *T*_max_ = 0.99
10775 measured reflections
1647 independent reflections
1170 reflections with *I* > 2σ(*I*)
*R*_int_ = 0.077

#### Refinement

*R*[*F* ^2^ > 2σ(*F* ^2^)] = 0.038
*wR*(*F* ^2^) = 0.087
*S* = 0.88
1647 reflections
164 parameters
H-atom parameters constrained
Δρ_max_ = 0.53 e Å^−3^
Δρ_min_ = −0.45 e Å^−3^

### 

Data collection: *COLLECT* (Nonius, 2001 ▶).; cell refinement: *DENZO*/*SCALEPACK* (Otwinowski & Minor, 1997 ▶); data reduction: *DENZO*/*SCALEPACK*; program(s) used to solve structure: *SIR92* (Altomare *et al.*, 1994 ▶); program(s) used to refine structure: *CRYSTALS* (Betteridge *et al.*, 2003 ▶); molecular graphics: *CAMERON* (Watkin *et al.*, 1996 ▶); software used to prepare material for publication: *CRYSTALS*.

## Supplementary Material

Crystal structure: contains datablocks global, I. DOI: 10.1107/S160053681001500X/lh5031sup1.cif

Structure factors: contains datablocks I. DOI: 10.1107/S160053681001500X/lh5031Isup2.hkl

Additional supplementary materials:  crystallographic information; 3D view; checkCIF report

## Figures and Tables

**Table 1:** Hydrogen-bond geometry (Å, °)

*D*—H⋯*A*	*D*—H	H⋯*A*	*D*⋯*A*	*D*—H⋯*A*
O15—H151⋯O1^i^	0.84	2.14	2.930 (4)	157
O15—H151⋯N7^i^	0.84	2.52	3.072 (4)	125

Symmetry code: (i) 
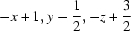
.

**Fig. 1.**
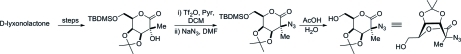
Synthetic Scheme

**Fig. 2.**
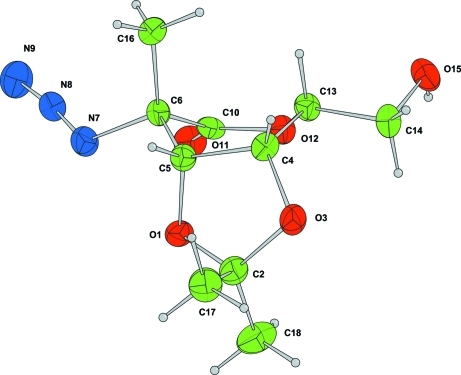
The title compound with displacement ellipsoids drawn at the 50% probability level. H atoms are shown as spheres of arbitary radius.

**Fig. 3.**
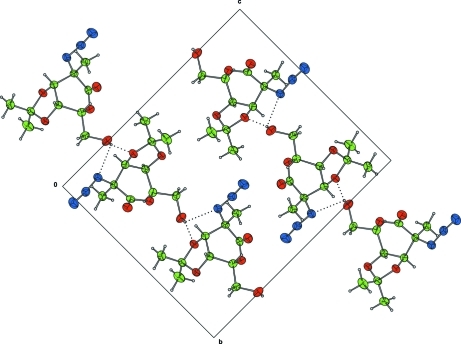
Packing diagram for the title compound projected along the a-axis. Hydrogen bonds are shown by dotted lines.

## supplementary crystallographic information

### Comment

Carbohydrates are a diverse set of chirons for the synthesis of complex amino acids and iminosugars (Lichtenthaler & Peters, 2004; Fechter et al., 1999; Fleet, 1989). 2-C-Methyl branched sugars constitute a class of rare sugars with chemotherapeutic potential (Rao et al., 2008; Jones et al., 2008; Booth et al., 2008) and can be used as building blocks in the synthesis of biologically active compounds (da Cruz et al., 2008; Hotchkiss, Kato et al., 2007; Soengas et al., 2005).

The azidolactone 3 (Fig. 1) would be a key intermediate for the synethsis of branched pyrrolidines, piperidines and prolines derived from D-lyxonolactone. Nucleophilic displacement of a triflate leaving group at the tertiary centre by azide was confirmed by X-ray crystallography to have proceeded with overall inversion of configuration (Booth et al. 2007; Hotchkiss, Jenkinson et al. 2007). The 6-membered lactone ring adopts a boat conformation, as is common with 3,4-O-isopropylidene-1,5-lactones (Baird et al., 1987; Bruce et al.,  1990; Punzo et al., 2005), with the larger azide group, rather than the methyl, in the bowsprit position (Fig. 2). The absolute configuration was determined from the use of D-lyxonolactone as the starting material. As is common with these materials the azide is non linear [N7 - N8 - N9 = 172.4 (3) °] (Chesterton et al., 2006), with the anisotropic atomic displacement parameter of the central atom lowered with respect to its neighbours. The compound exists as hydrogen bonded chains of molecules running parallel to the b-axis (Fig. 3). The hydrogen bond is bifurcated. Only classical hydrogen bonding is considered.

### Experimental

The title compound was recrystallised by slow evaporation from a mixture of diethyl ether and cyclohexane: m.p. 397-403 K, [α]_D_^25^ +112.4 (*c*, 1.145 in CHCl_3_).

### Refinement

In the absence of significant anomalous scattering, Friedel pairs were merged and the absolute configuration was assigned from the use of D-lyonolactone as the starting material.

The H atoms were all located in a difference map, but those attached to carbon atoms were repositioned geometrically. The H atoms were initially refined with soft restraints on the bond lengths and angles to regularize their geometry (C—H in the range 0.93–0.98, O—H = 0.82 Å) and *U*_iso_(H) (in the range 1.2–1.5 times *U*_eq_ of the parent atom), after which the positions were refined with riding constraints.

### Crystal data

**Table:**

C_10_H_15_N_3_O_5_	*F*(000) = 544
*M**_r_* = 257.25	*D*_x_ = 1.377 Mg m^−^^3^
Orthorhombic, *P*2_1_2_1_2_1_	Mo *K*α radiation, λ = 0.71073 Å
Hall symbol: P 2ac 2ab	Cell parameters from 1637 reflections
*a* = 5.9481 (3) Å	θ = 5–27°
*b* = 13.3427 (7) Å	µ = 0.11 mm^−^^1^
*c* = 15.6351 (9) Å	*T* = 150 K
*V* = 1240.86 (12) Å^3^	Plate, colourless
*Z* = 4	0.20 × 0.15 × 0.05 mm

### Data collection

**Table:**

Nonius KappaCCD diffractometer	1170 reflections with *I* > 2σ(*I*)
graphite	*R*_int_ = 0.077
ω scans	θ_max_ = 27.5°, θ_min_ = 5.2°
Absorption correction: multi-scan (*DENZO*/*SCALEPACK*; Otwinowski & Minor, 1997)	*h* = −7→7
*T*_min_ = 0.89, *T*_max_ = 0.99	*k* = −17→17
10775 measured reflections	*l* = −20→20
1647 independent reflections	

### Refinement

**Table:**

Refinement on *F*^2^	Hydrogen site location: inferred from neighbouring sites
Least-squares matrix: full	H-atom parameters constrained
*R*[*F*^2^ > 2σ(*F*^2^)] = 0.038	Method = Modified Sheldrick *w* = 1/[σ^2^(*F*^2^) + ( 0.05*P*)^2^ + 0.16*P*], where *P* = [max(*F*_o_^2^,0) + 2*F*_c_^2^]/3
*wR*(*F*^2^) = 0.087	(Δ/σ)_max_ = 0.0003
*S* = 0.88	Δρ_max_ = 0.53 e Å^−^^3^
1647 reflections	Δρ_min_ = −0.45 e Å^−^^3^
164 parameters	Extinction correction: Larson (1970), Equation 22
0 restraints	Extinction coefficient: 460 (60)
Primary atom site location: structure-invariant direct methods	

### Fractional atomic coordinates and isotropic or equivalent isotropic displacement parameters (Å^2^)

**Table:**

	*x*	*y*	*z*	*U*_iso_*/*U*_eq_	
O1	0.4977 (3)	0.87439 (12)	0.79200 (10)	0.0276	
C2	0.5736 (5)	0.85325 (19)	0.87769 (16)	0.0297	
O3	0.7326 (3)	0.77432 (13)	0.86551 (10)	0.0334	
C4	0.8307 (4)	0.78267 (17)	0.78210 (15)	0.0258	
C5	0.6901 (4)	0.86484 (17)	0.73767 (14)	0.0250	
C6	0.6110 (4)	0.83522 (18)	0.64929 (15)	0.0247	
N7	0.4436 (4)	0.91275 (16)	0.62475 (14)	0.0317	
N8	0.3742 (4)	0.90581 (16)	0.55031 (15)	0.0313	
N9	0.2976 (4)	0.90888 (18)	0.48383 (15)	0.0443	
C10	0.4914 (4)	0.73333 (18)	0.65603 (15)	0.0243	
O11	0.3123 (3)	0.71606 (13)	0.62348 (11)	0.0323	
O12	0.5913 (3)	0.66364 (12)	0.70449 (11)	0.0256	
C13	0.8169 (4)	0.68186 (17)	0.73740 (16)	0.0250	
C14	0.8716 (5)	0.59403 (17)	0.79413 (17)	0.0309	
O15	0.8866 (3)	0.50433 (11)	0.74599 (11)	0.0351	
C16	0.8056 (4)	0.83600 (19)	0.58502 (16)	0.0303	
C17	0.6857 (5)	0.9437 (2)	0.91665 (18)	0.0385	
C18	0.3762 (5)	0.8142 (2)	0.92680 (19)	0.0459	
H41	0.9905	0.8032	0.7872	0.0311*	
H51	0.7740	0.9284	0.7350	0.0310*	
H131	0.9235	0.6813	0.6888	0.0288*	
H141	1.0180	0.6075	0.8209	0.0398*	
H142	0.7552	0.5873	0.8388	0.0391*	
H161	0.7461	0.8167	0.5292	0.0461*	
H162	0.8707	0.9027	0.5818	0.0463*	
H163	0.9219	0.7893	0.6024	0.0460*	
H172	0.7391	0.9258	0.9730	0.0598*	
H171	0.5743	0.9972	0.9206	0.0603*	
H173	0.8113	0.9635	0.8797	0.0603*	
H182	0.4260	0.7957	0.9845	0.0690*	
H181	0.2604	0.8655	0.9297	0.0694*	
H183	0.3174	0.7559	0.8975	0.0688*	
H151	0.7591	0.4778	0.7453	0.0532*	

### Atomic displacement parameters (Å^2^)

**Table:**

	*U*^11^	*U*^22^	*U*^33^	*U*^12^	*U*^13^	*U*^23^
O1	0.0272 (9)	0.0348 (9)	0.0207 (8)	0.0036 (8)	−0.0008 (8)	0.0009 (7)
C2	0.0358 (14)	0.0323 (13)	0.0209 (12)	0.0025 (12)	−0.0027 (11)	−0.0011 (12)
O3	0.0480 (11)	0.0305 (9)	0.0217 (9)	0.0089 (9)	−0.0031 (8)	0.0004 (8)
C4	0.0254 (13)	0.0278 (12)	0.0241 (13)	−0.0041 (11)	−0.0031 (10)	0.0007 (11)
C5	0.0249 (12)	0.0250 (12)	0.0252 (12)	0.0006 (10)	−0.0006 (11)	0.0016 (11)
C6	0.0253 (12)	0.0255 (12)	0.0233 (13)	0.0057 (11)	−0.0011 (11)	0.0031 (10)
N7	0.0361 (12)	0.0337 (11)	0.0253 (11)	0.0090 (10)	−0.0035 (10)	−0.0003 (10)
N8	0.0309 (12)	0.0300 (11)	0.0330 (13)	0.0066 (10)	0.0013 (11)	0.0030 (11)
N9	0.0446 (14)	0.0544 (16)	0.0340 (14)	0.0089 (13)	−0.0105 (12)	0.0052 (12)
C10	0.0210 (12)	0.0297 (13)	0.0221 (11)	0.0039 (11)	0.0023 (11)	−0.0047 (11)
O11	0.0255 (9)	0.0391 (10)	0.0322 (10)	−0.0029 (9)	−0.0047 (8)	−0.0022 (9)
O12	0.0228 (8)	0.0246 (8)	0.0293 (9)	−0.0005 (7)	−0.0023 (7)	0.0012 (8)
C13	0.0192 (11)	0.0267 (12)	0.0292 (14)	0.0008 (10)	−0.0021 (11)	0.0020 (11)
C14	0.0327 (14)	0.0247 (12)	0.0351 (14)	0.0018 (12)	−0.0044 (12)	0.0045 (12)
O15	0.0297 (9)	0.0248 (9)	0.0509 (12)	0.0035 (8)	0.0037 (9)	0.0003 (9)
C16	0.0318 (13)	0.0317 (13)	0.0274 (13)	0.0010 (12)	0.0036 (11)	0.0049 (11)
C17	0.0484 (17)	0.0374 (15)	0.0297 (14)	0.0015 (14)	−0.0060 (14)	−0.0059 (12)
C18	0.0446 (17)	0.063 (2)	0.0300 (16)	−0.0052 (15)	0.0033 (13)	0.0041 (15)

### Geometric parameters (Å, °)

**Table:**

O1—C2	1.442 (3)	C10—O12	1.338 (3)
O1—C5	1.431 (3)	O12—C13	1.458 (3)
C2—O3	1.428 (3)	C13—C14	1.505 (3)
C2—C17	1.508 (4)	C13—H131	0.989
C2—C18	1.496 (4)	C14—O15	1.417 (3)
O3—C4	1.433 (3)	C14—H141	0.983
C4—C5	1.544 (3)	C14—H142	0.987
C4—C13	1.518 (3)	O15—H151	0.837
C4—H41	0.993	C16—H161	0.977
C5—C6	1.512 (3)	C16—H162	0.972
C5—H51	0.985	C16—H163	0.969
C6—N7	1.486 (3)	C17—H172	0.967
C6—C10	1.538 (3)	C17—H171	0.976
C6—C16	1.533 (3)	C17—H173	0.981
N7—N8	1.238 (3)	C18—H182	0.981
N8—N9	1.136 (3)	C18—H181	0.972
C10—O11	1.203 (3)	C18—H183	0.969
			
C2—O1—C5	106.47 (18)	C4—C13—O12	111.08 (19)
O1—C2—O3	103.17 (18)	C4—C13—C14	114.0 (2)
O1—C2—C17	110.9 (2)	O12—C13—C14	106.09 (19)
O3—C2—C17	110.6 (2)	C4—C13—H131	109.0
O1—C2—C18	107.4 (2)	O12—C13—H131	108.6
O3—C2—C18	109.4 (2)	C14—C13—H131	108.0
C17—C2—C18	114.7 (2)	C13—C14—O15	111.0 (2)
C2—O3—C4	109.49 (17)	C13—C14—H141	107.5
O3—C4—C5	104.14 (19)	O15—C14—H141	109.0
O3—C4—C13	109.17 (18)	C13—C14—H142	109.6
C5—C4—C13	113.14 (19)	O15—C14—H142	110.1
O3—C4—H41	109.8	H141—C14—H142	109.7
C5—C4—H41	111.0	C14—O15—H151	107.9
C13—C4—H41	109.5	C6—C16—H161	108.1
C4—C5—O1	103.26 (17)	C6—C16—H162	110.0
C4—C5—C6	113.20 (19)	H161—C16—H162	109.8
O1—C5—C6	108.46 (18)	C6—C16—H163	110.5
C4—C5—H51	110.9	H161—C16—H163	109.9
O1—C5—H51	110.7	H162—C16—H163	108.6
C6—C5—H51	110.1	C2—C17—H172	108.4
C5—C6—N7	105.21 (19)	C2—C17—H171	108.1
C5—C6—C10	108.20 (19)	H172—C17—H171	110.3
N7—C6—C10	108.84 (18)	C2—C17—H173	108.3
C5—C6—C16	111.2 (2)	H172—C17—H173	110.7
N7—C6—C16	109.39 (19)	H171—C17—H173	111.0
C10—C6—C16	113.6 (2)	C2—C18—H182	108.8
C6—N7—N8	114.5 (2)	C2—C18—H181	109.6
N7—N8—N9	172.4 (3)	H182—C18—H181	110.4
C6—C10—O11	123.4 (2)	C2—C18—H183	108.6
C6—C10—O12	116.6 (2)	H182—C18—H183	110.0
O11—C10—O12	120.0 (2)	H181—C18—H183	109.4
C10—O12—C13	119.50 (19)		

### Hydrogen-bond geometry (Å, °)

**Table:**

*D*—H···*A*	*D*—H	H···*A*	*D*···*A*	*D*—H···*A*
C5—H51···O15^i^	0.99	2.28	3.141 (4)	146
C13—H131···O11^ii^	0.99	2.57	3.473 (4)	152
C16—H161···O11^iii^	0.98	2.46	3.333 (4)	149
C16—H163···O11^ii^	0.97	2.54	3.465 (4)	159
O15—H151···O1^iv^	0.84	2.14	2.930 (4)	157
O15—H151···N7^iv^	0.84	2.52	3.072 (4)	125

Symmetry codes: (i) −*x*+2, *y*+1/2, −*z*+3/2; (ii) *x*+1, *y*, *z*; (iii) *x*+1/2, −*y*+3/2, −*z*+1; (iv) −*x*+1, *y*−1/2, −*z*+3/2.
